# The Role of Natural Killer Cells in Oncolytic Virotherapy: Friends or Foes?

**DOI:** 10.3390/vaccines12070721

**Published:** 2024-06-28

**Authors:** Michael L. Franks, Ju-Hyun An, Jianmei W. Leavenworth

**Affiliations:** 1 Department of Neurosurgery, University of Alabama at Birmingham, Birmingham, AL 35233, USA; mlfranks@uab.edu (M.L.F.);; 2 Graduate Biomedical Sciences Program, University of Alabama at Birmingham, Birmingham, AL 35294, USA; 3 The O’Neal Comprehensive Cancer Center, University of Alabama at Birmingham, Birmingham, AL 35294, USA; 4 Department of Microbiology, University of Alabama at Birmingham, Birmingham, AL 35294, USA

**Keywords:** oncolytic virotherapy, oncolytic virus, natural killer cells, antiviral response, antitumor response

## Abstract

Oncolytic virotherapy (OVT) has emerged as a promising cancer immunotherapy, and is capable of potentiating other immunotherapies due to its capacity to increase tumor immunogenicity and to boost host antitumor immunity. Natural killer (NK) cells are a critical cellular component for mediating the antitumor response, but hold a mixed reputation for their role in mediating the therapeutic efficacy of OVT. This review will discuss the pros and cons of how NK cells impact OVT, and how to harness this knowledge for the development of effective strategies that could modulate NK cells to improve OVT-based therapeutic outcomes.

## 1. Introduction

Immunotherapies, such as immune checkpoint blockade (ICB) and chimeric antigen receptor (CAR)-engineered cellular therapy, have shown promise in cancer patients. However, the immunosuppressive tumor microenvironment (TME) inherent in many types of cancer often represents a huge hurdle to favorable therapeutic outcomes [1]. Oncolytic virotherapy (OVT) has been proposed as a strategy to address this barrier. OVT utilizes viruses that preferentially infect and kill cancer cells while promoting a subsequent antitumor immune response. This process reshapes the TME and increases the immunogenicity of a tumor, converting immunosuppressive “cold” tumors into immunostimulatory “hot” tumors. The immune-associated mechanisms underlying this conversion have been extensively reviewed elsewhere [2,3,4]. Interestingly, studies of the impact of OVT on the host immune response have revealed that natural killer (NK) cells play a complicated role in OVT-based therapeutic outcomes. NK cells are integral to the innate immune response against both viral infection and tumors. Unfortunately, the antiviral response of NK cells has been shown to limit the spread of oncolytic viruses (OVs), often impeding their therapeutic efficacy. On the other hand, in some OVTs, NK cells have demonstrably contributed to their therapeutic efficacy, and several studies have successfully utilized the potent antitumor response of NK cells by combining OVT with other immunotherapies. This review will focus on the multifaceted aspects of NK cells in OVT, and the strategies that have successfully utilized NK cells to improve OVT efficacy against cancer.

## 2. Immune Response to Cancer

The immune surveillance mechanism against cancer is often explained by the cancer-immunity (CI) cycle [5,6,7]. The CI cycle was first proposed in the context of the T-cell-mediated antitumor response, illustrating the cyclic processes starting from the cancer’s antigen release to the acquisition of adaptive immunity against cancer [7]. The T-cell-focused CI cycle is initiated by antigen-presenting cells (APCs), such as dendritic cells (DCs), which uptake and present tumor antigens, thereby priming and activating T-cells. The activated T-cells then migrate, infiltrate the tumor and kill the cancer cells, consequently leading to the release of more tumor antigens. Since the T-cell CI cycle requires the presence of initial antitumor immune responses, its initiation is highly dependent on the immunological milieu of the tumor parenchyma, referred to as tumor immunotypes, including the immune desert, immune excluded, and immune inflamed [6]. Immune-desert tumors are defined as tumors devoid of tumor-infiltrating lymphocytes (TILs), with prevalent WNT/β-catenin signaling and include many neuroendocrine tumors [8,9,10]. Immune-excluded tumors are characterized by abundant immune-suppressive cell populations located within or proximal to the tumor site, including tumor-associated macrophages (TAMs), myeloid-derived suppressor cells (MDSCs), and regulatory T-cells (Tregs). These cells release immunosuppressive cytokines, such as interleukin (IL)-10, IL-35, and transforming growth factor (TGF)-β [11,12,13,14,15], consequentially leading to the exhaustion and poor tumor infiltration of the immune effector cells and failure of tumor clearance. In contrast, immune-inflamed tumors are characterized by a TME rich in interferon-gamma (IFN-γ), a high tumor mutational burden, a large population of TILs, and an elevated expression of major histocompatibility complex (MHC) class I/II in tumor cells [16,17,18,19,20]. Recent findings have revealed that NK cells play a pivotal role in shaping the immune-inflamed immunotype by not only directly killing and debulking the cancer mass, but also by secreting proinflammatory cytokines [5]. Most importantly, IFN-γ promotes the activation and maturation of APCs, invigorating the intervention of the adaptive immune system against cancers and amplifying the CI cycle [21,22,23,24].

The failure of immune surveillance that is involved in each step of the CI cycle often leads to cancer growth and progression, which proceeds through three sequential phases, including elimination, equilibrium, and escape, referred to as cancer immunoediting [25]. During elimination, the growing tumor stresses the tissues surrounding it, creating a proinflammatory environment, and prompting an innate immune response. NK cells are cytotoxic lymphocytes that are integral to the innate immune response to cancer. They detect and kill tumor cells as well as release IFN-γ, which promotes APCs to upregulate MHCI/II expression [22,26]. APCs migrate to the draining lymph node, where the selection and recruitment of adaptive immune cells, such as CD4^+^ or CD8^+^ T-cells, specific to tumor antigens can begin. During the elimination phase, cancer cells are effectively killed by NK and cytotoxic T-cells, and the cancer can be resolved at this point [25]. The equilibrium phase is entered into if the immune system is not able to eradicate the cancer entirely. During this phase, the growth of the cancer is controlled by the immune system, and the cancer cells undergo a natural selection process, acquiring resistance to antitumor immunity [25,27]. The escape phase occurs when the cancer cells have acquired enough resistance to grow and spread beyond the immune surveillance [25,27]. The methods by which cancer cells attain resistance to antitumor immunity are varied, including the release of suppressive cytokines to inhibit immune cell activity, the downregulation of antigen presentation, the upregulation of inhibitory ligands, and the attraction of immune-suppressive regulatory cells, which have been extensively described [28].

## 3. Cancer Immunotherapies

Cancer immunotherapies have been developed to modulate the immune response to cancer, facilitating re-entry into the elimination phase and amplifying the CI cycle [5,6,7]. ICB, cancer-targeting monoclonal antibodies, and CAR-engineered cellular therapy are the major classes of cancer immunotherapies. ICBs are antibodies that target either the inhibitory receptors on immune effector cells, or the inhibitory ligands expressed on tumor cells and, in some cases, immune cells. Inhibitory receptors, such as programmed cell death protein 1 (PD-1) and cytotoxic T lymphocyte-associated protein 4 (CTLA-4), are often upregulated in activated lymphocytes to prevent hyperactivation, or are expressed on immunosuppressive cells, like Tregs. Many cancers have been reported to upregulate inhibitory ligands, such as programmed cell death ligand 1 (PD-L1) and PD-L2, facilitating their escape from immune surveillance [29]. ICBs competitively inhibit the binding of inhibitory receptors to their cognate ligands, enabling cytotoxic immune cells to attack cancer cells [9,10]. In addition to ICBs, monoclonal antibodies designed to specifically target cancer cells have also been shown to promote antitumor responses. Cancer-bound antibodies facilitate the antibody-dependent cellular cytotoxicity (ADCC) of macrophages and NK cells that express CD16, also known as FcγRIII [30,31,32]. CAR-T and CAR-NK cells are cytotoxic lymphocytes armed with CARs specific to the tumor antigens expressed by their target cancer cells. CARs ensure the engagement of cytotoxic lymphocytes against cancer cells, launching an antigen-specific response with an emphasis on cell-to-cell cytotoxic killing [33,34].

Immunotherapies have changed the paradigm for the treatment of cancer patients with advanced disease stages. However, their efficacy is highly dependent upon the immune phenotype of the tumor. Immunotherapies, such as ICBs, have been most successful in immune-inflamed tumors. In such tumors, ICBs block the inhibitory signals, enhancing the tumor-killing efficacy of tumor-infiltrating immune cells. In contrast, ICBs have been less effective on tumors with immune-excluded and immune-desert immunotypes, as in these tumors, the effector cells are already exhausted or can barely infiltrate the tumor site. To overcome this barrier, strategies that are designed to shift the immune phenotype of a cancer towards immune-inflamed status to improve the efficacy of cancer immunotherapies are being developed, and one such strategy is OVT.

## 4. Oncolytic Virotherapy

### 4.1. Introduction to Oncolytic Viruses

OVT utilizes viruses that preferentially infect and replicate in cancer cells compared to non-cancer cells [35]. The clearance of cancer cells during viral infections was noted as far back as the early 20th century. In 1912, tumor regression was noted in a cervical cancer patient after receiving the rabies vaccine, which contains an attenuated version of the rabies virus [36]. Tumor regression has also been seen in response to measles, polio, smallpox, adenovirus, vestibular stomatitis virus (VSV), and herpes simplex virus 1 (HSV-1), among others [37,38,39,40,41,42]. Cancer cells are favorable hosts for viral infection because their cellular machinery for DNA replication is highly upregulated to support rapid proliferation, which facilitates viral DNA replication upon infection. Additionally, many cancer cells exhibit impaired protein kinase R (PKR) pathway activity, which has antiviral defense mechanisms, by limiting protein synthesis and inducing apoptosis upon viral genome detection, making tumor cells more vulnerable to viral infection [43]. In addition, some cancers are known to have dysfunctional type 1 interferon pathways, increasing their susceptibility to viruses [44].

OVs kill cancer cells and promote antitumor immune responses. The replication of OVs in cancer cells induces the lysis of tumor cells, leading to the release of pathogen-associated molecular pattern molecules (PAMPs), damage-associated molecular pattern molecules (DAMPs), type 1 interferons, viral antigens, and tumor antigens into the TME. All these factors subsequently provoke a robust immune response [45,46,47]. OVT-mediated innate immune responses prompt the recruitment and maturation of DCs [48,49,50,51,52]. As professional APCs, DCs uptake, process, and present tumor antigens to T-cells, facilitating adaptive immunity against cancer, in a process called epitope spreading [47,53,54]. Interestingly, recent research has demonstrated that pre-existing immunity to viruses also contributes to developing antitumor adaptive immunity [55,56]. In the murine colon adenocarcinoma MC38 model, the bystander T-cells specific to the lymphocytic choriomeningitis virus (LCMV) within the TME exhibited the specific killing of infected tumor cells, substantially contributing to DC-mediated cancer epitope spreading and acquisition of antitumor immunity [56]. Similarly, another study also demonstrated that the therapeutic efficacy of oncolytic Newcastle disease virus (NDV) can be potentiated by pre-existing immunity to NDV [55]. Taken together, these studies suggest that antiviral immune responses to OVT potentially potentiate antitumor responses.

### 4.2. Oncolytic Viruses for Cancer Immunotherapy

Several different viruses have exhibited prominent anticancer efficacy, including the adenovirus [57], reovirus [58], VSV [41], vaccinia virus (VV) [59], and HSV [60]. The use of oncolytic adenoviruses (oAds) is one of the earliest forms of OVT to enter clinical trials and has undergone extensive research. oAds can infect a wide range of cancer cells and release highly immunogenic viral components [61]. Cancer cells killed by oAds exhibit features of necrosis and autophagy [62], and release potent immunostimulants, classified as DAMPs, such as ATP, calreticulin, and heat shock protein 70 [62,63,64]. Reovirus is a non-encapsulated double-stranded RNA virus, and its lytic cycle can be accelerated by Ras signaling activation. This property makes the reovirus ideal for targeting cancers harboring constitutively active Ras mutations [65]. Additionally, DCs exposed to reovirus have been shown to drive potent antitumor immune responses [66]. VSV is a non-segmented RNA virus in the family Rhabdoviridae. Due to the absence of an immunological memory against VSV in the majority of human population, oncolytic recombinant VSVs have exhibited high tolerability when administered [67]. Alongside this property, the high viral titer and tumor trophism enable the systemic administration of VSV, making it a promising therapeutic option [67,68,69]. Oncolytic VV is distinguished by its hypoxic TME tolerability and antigenic variety [70,71,72]. The properties of each OV have been extensively discussed elsewhere [2]. We will focus on oncolytic HSV (oHSV) in the following sections.

### 4.3. Genetic Engineering in OVT

OVs have been genetically engineered to improve their safety and efficacy. For example, HSV-1 has been genetically engineered to improve its tumor-killing efficacy and safety profile. HSV-1 is a double-stranded DNA virus, characterized by a large linear genome (~25 kb), which allows HSV-1 to accommodate relatively large genetic modifications, such as transgene insertions for improving its anticancer efficacy [73]. Arming oHSV-1 through the insertion of proinflammatory cytokines, including granulocyte–macrophage colony-stimulating factor (GM-CSF) [74], IL-12 [75], and IL-15 [76,77], has improved its antitumor efficacy, and lacZ insertion has allowed for tracking viral distribution and infection [78]. Likewise, OVs can be armed with inhibitors targeting immune checkpoint receptors, such as anti-PD-1 single-chain variable fragment (scFv) [79,80] and anti-CTLA-4 scFv [81], to reduce the immunosuppressive milieu of a tumor. In addition, to facilitate the engagement of the adaptive immune system, DNA viruses can be modified to express tumor-associated antigens (TAAs), such as melanoma-derived tumor antigen gp100 [82], carcinoembryonic antigen (CEA) [83], glycoprotein oncofetal tumor antigen (5T4) [84], and lung cancer antigen (EphA2) [85].

In terms of the safety features of OVs, the initial roadblock for OVT is the off-target effects that deteriorate normal tissues. An example of genetic engineering that has improved the safety of OVT is the deletion of thymidine kinase in oHSV. Thymidine kinase plays a pivotal role in de novo synthesis of nucleic acids for viral genome replication, making it indispensable for oHSV multiplication [86]. While oHSVs with thymidine kinase mutations cannot replicate in non-proliferative cells, which are deficient in DNA replication machinery, they are able to readily infect and multiply in proliferative cells by exploiting the thymidine kinase of host cells [73]. In the context of OVT against brain tumors, employing the thymidine kinase knockout strategy mitigates the risk of infecting healthy neural tissues that are typically quiescent, while enabling oHSVs to selectively target highly proliferative cancer cells [87]. The *γ34.5* gene is another common target for deletion in oHSV. The *γ34.5* gene is a key factor of HSV-1-induced neurovirulence and is known to inhibit the aforementioned PKR pathway, thereby facilitating viral replication in host cells. The deletion of this gene substantially reduces the risk of oHSV-mediated neurovirulence by preventing viral protein synthesis in healthy cells, while maintaining its competence in cells with impaired PKR function, such as cancer cells [88,89].

The above-described genetic modifications have given oHSV a superior safety profile, making it an ideal candidate for treating neuronal brain cancers. A genetically engineered oHSV, which incorporates the deletion of thymidine kinase and ectopic IL-12 expression, has exhibited beneficial therapeutic outcomes against a variety of brain malignancies in preclinical trials [90,91,92]. IL-12 promotes anticancer immune responses by stimulating immune effector cells to directly target cancer cells via cytotoxicity or by secreting inflammatory cytokines [93,94,95,96,97]. Furthermore, genetically engineered oHSVs have shown superior therapeutic outcomes in clinical trials, including talimogene laherparepvec (T-VEC) for melanoma, the first OVT approved by the FDA [40]. T-VEC is a genetically engineered oHSV harboring the deletion of both *γ34.5* and *Herpes_US12* genes. The latter is known to inhibit antigenic peptide transportation to MHCI in infected cells [89,98]. In addition, proinflammatory cytokine GM-CSF has been introduced to facilitate DC recruitment and T-cell priming [40,99,100]. In clinical trials of T-VEC against cutaneous head and neck melanoma, 16% of the T-VEC-treated patients achieved a durable response rate (DRR), while the control group treated with GM-CSF alone only had a 2% DRR in a landmark phase 3 clinical trial [101,102]. In Austria, Switzerland, and Germany, T-VEC therapy has reported a complete response rate of 43.2% in melanoma patients [103]. G47Δ, another genetically engineered oHSV, has also shown encouraging therapeutic outcomes in clinical trials. The deletion of the *α47* gene in G47Δ is designed to increase MHCI expression in infected cells [104]. In a phase 2 clinical trial, G47Δ achieved a superior 1-year survival rate in patients with recurring glioblastoma multiforme (GBM) of 92.3%, which is a substantial improvement over the 14% seen in patients who received standard chemo–radio therapy [105,106]. This result led to the approval of G47Δ as a treatment for malignant glioma in Japan. While not yet approved, clinical trials for another engineered oHSV, named G207, have demonstrated promising therapeutic outcomes, with a median survival of 12.2 months versus 5.6 months for the current standard of care received by pediatric patients with high-grade glioma [60]. G207 has also been shown to improve immune infiltration into GBM tumors [107] and is able to further extend survival in adult patients with recurring GBM when combined with radiotherapy [108]. Lastly, CAN-3110, an engineered oHSV designed to express the *γ34.5* gene under the tumor-specific promoter, has shown enhanced viral replication upon infection and the improved survival of high-grade glioma patients [109]. In summary, genetic engineering technologies in the past decades have significantly improved the safety and efficacy of oncolytic viruses, thereby leading to the development of promising OVs against cancer.

### 4.4. The Controversial Role of the Host Immune System in OVT

**Figure 1:**
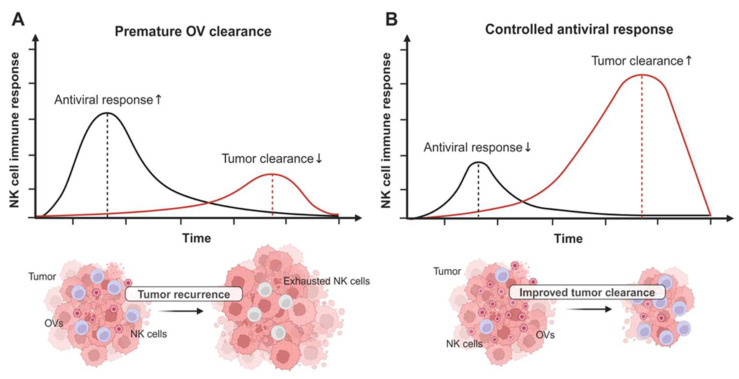
Premature OV clearance dampens long-term therapeutic outcomes of OVT. (**A**) Antiviral response-mediated early OV clearance leads to poor tumor rejection and tumor recurrence. (**B**) Controlled antiviral response allows OV to spread within a tumor, resulting in improved therapeutic outcomes.

As described above, the host immune response to OVT is crucial to its therapeutic efficacy. The infection and subsequent lysis of tumor cells by oncolytic viruses lead to the release of proinflammatory molecule cascades into the TME, including type 1 interferons, DAMPs, PAMPs, IL-18, IL-1β, and also tumor antigens [53,110,111,112,113,114], generating robust antiviral and antitumor immune responses. In the context of OVT, antiviral responses have been believed to be detrimental, as they limit the spread of OVs. However, further studies have demonstrated that the antiviral response is necessary to prime both the innate and adaptive antitumor responses [47]. As part of the innate immune system, the antiviral responses mediated by NK cells, compared to other cytotoxic lymphocytes, occur in the early phase of OVT [115], and have been shown to impede OVT efficacy by killing infected cells at early timepoints, limiting the reservoirs of viral replication [115] (Figure 1A). In contrast, NK cells have been shown to contribute to the OV-mediated antitumor response in a variety of cancers [116,117,118], likely when the antiviral response is controlled (Figure 1B). Collectively, NK cells could possibly lead to controversial therapeutic outcomes, depending on the balance between their antiviral and antitumor properties (Figure 1). Understanding both antitumor and antiviral immune responses in the context of OVT will shed light on how to design OVTs that utilize the maximal potential of NK cells.

## 5. NK Cell Biology

### 5.1. NK Cell Antitumor Responses

NK cells play a pivotal role in immune surveillance and are the primary defense against abnormal cells in the body, including infected, stressed, and neoplastic cells. Unlike cytotoxic T-cells, NK cells are not antigen specific; instead, they detect abnormal cells via a set of inhibitory and activating receptors. The functional activity of NK cells is determined by the balance between the signals emanating from these receptors, which are clustered in the immunological synapses of the NK cell, and its target [119]. Under homeostatic conditions, NK cells maintain basal levels of activation through the presentation of natural cytotoxicity receptors (NCRs) and other activation receptors on their surface, including NKp46, NKp30, NKp44, NKG2D (an NKG2 family member of C-type lectin-like receptor), and toll-like receptors (TLRs). This baseline receptor repertoire enables them to promptly respond to abnormal cells [120,121,122,123,124]. NKG2D and NCRs are the receptors that play an important role in the NK cell-mediated antitumor response. These receptors have been shown to be crucial in preventing early tumor formation by recognizing stress-induced ligands that are expressed on different types of cancer cells [125,126,127], such as MHC class I-related polypeptide sequence A (MICA) [127,128,129], natural killer cell cytotoxicity receptor 3 ligand 1 (NCR3LG1), and BCL2-associated athanogene 6 (BAG6) [130,131]. Inhibitory receptors present on NK cells, including killer immunoglobulin-like receptors (KIR), bind to MHCI molecules, thereby sustaining immune tolerance for healthy cells and preventing an autoimmune response. Tumor cells often downregulate MHCI expression, making them vulnerable to NK cell-mediated cytotoxicity. Another major mechanism of cancer recognition by NK cells is the detection of heat shock proteins (HSPs). HSPs are highly conserved molecular chaperones that prevent protein misfolding and aggregation under stressful conditions, and help transport antigenic peptides to MHCI/II molecules [132,133,134]. For example, 70 kDa heat shock proteins (HSP70s), which are highly upregulated in cancer, support tumor growth and metastasis by disrupting various pathways, leading to reduced apoptosis and increased proliferation and angiogenesis [135,136]. Membrane-bound HSP70s on cancer cells are recognized by the C-type lectin receptor CD94 on NK cells, exerting their antitumor activities [137,138,139]. Additionally, HSP70s upregulate MICA expression, facilitating NK cell-mediated tumor clearance via NKG2D [140].

**Figure 2:**
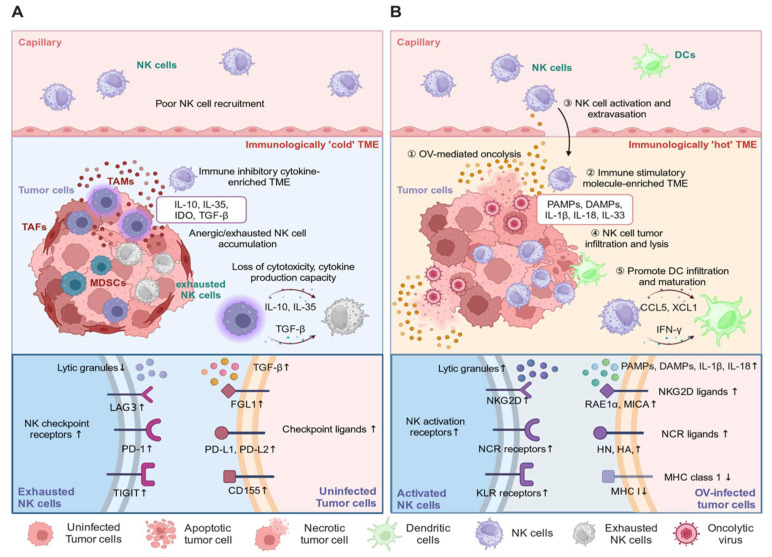
OVT promotes NK cell infiltration and tumor killing. (**A**) The immune-suppressive TME upregulates checkpoint receptors and ligands on NK and tumor cells, respectively, thereby inducing NK cell dysfunction and exhaustion. (**B**) OVT converts the immunologically “cold” TME to the “hot” condition, promoting NK cell infiltration and tumor lysis.

The killing capacity of NK cells against cancer cells within the TME is impeded by a variety of immune-suppressive mechanisms that drive the NK cell signaling balance towards inhibition (Figure 2A). NK cell gene expression is rapidly altered upon entering a tumor, resulting in impaired cytokine and chemokine production, as well as granzyme secretion. This alteration impedes the antitumor functionality of NK cells and their capacity to recruit and activate DCs, which is thought to be caused, in part, by immunosuppressive cytokines and hypoxic conditions within the TME [141]. The TAMs and MDSCs often present within the TME secrete immune-suppressive cytokines, such as IL-10 and TGF-β, which downregulate the expression of activation receptors and suppress the lytic granule release of NK cells [142]. Additionally, interactions between NK cells and extracellular matrix (ECM) components, such as collagen, fibronectin, laminin, and elastin, promote the differentiation of NK cells, downregulating NK cell cytotoxicity and upregulating cytokine production [143,144]. This functional transition impairs NK cell-mediated tumor clearance [143,144,145]. Fibronectin was recently identified as a ligand of leukocyte immunoglobulin-like receptor subfamily B member 4 (LILRB4), one of NK cells inhibitory receptors, and their interaction attenuates NK cell killing of Lewis lung carcinoma cells [145]. The ECM is also a structural barrier to NK cell infiltration [146]. Treating melanoma-bearing mice with losartan and dihydroxybenzoic acid (DHB), which impair collagen synthesis and assembly, restores NK cell antitumor functionality and overall mice survival [143]. One strategy designed to cope with the ECM barrier is the oVV-Hyal1, an oncolytic VV-expressing hyaluronidase, which substantially degrades ECM components, such as collagen I/III, elastin, fibronectin, and hyaluronic acid, improving lymphocyte infiltration and antitumor efficacy [147]. Also, indoleamine 2,3-dioxygenase (IDO) in the TME leads to the depletion of tryptophan and the accumulation of L-kynurenine, inducing NK cell exhaustion and dysfunction [148], accompanied by the downregulation of NKG2D and NKp30 [148]. Interestingly, NKG2D ligands often shed into the extracellular milieu of the TME, which can be facilitated by various proteases, including A disintegrin and metalloprotease (ADAM)-10, ADAM-17, endoplasmic reticulum protein 5 (Erp5), and matrix metallopeptidase 14 (MMP14) [149,150,151]. Interactions with the soluble forms of NKG2D ligands leads to the internalization of NKG2D, thereby impairing NK cell antitumor activity, which is another mechanism underlying NK cell paralysis in a tumor [152,153,154,155].

### 5.2. NK Cell Antiviral Responses

NK cells also play a critical role in controlling viral infections, as the loss of NK cell functionality results in severe and even fatal infection from relatively benign viruses, such as Epstein Barr virus (EBV), HSV, and varicella zoster virus (VZV). There is an increased incidence of human papilloma virus (HPV)- and EBV-induced cancers [156,157,158], and influenza infection has exhibited higher lethality in a mouse model bearing a loss of function of *Ncr1*, which encodes NKp46 [120]. Similar to the way that NK cells target tumor cells, the detection and killing of infected cells are governed by the balance between the activating and inhibitory signals received by NK cells. The strength of these signals is determined by the following mechanisms: (1) the interaction of PAMPs and DAMPs with TLRs [159,160], (2) proinflammatory cytokine stimulation [161] and, finally, (3) the interplay of inhibitory and activation receptors on NK cells with their cognate ligands [120,162,163]. While crucial for host defense against viral infections, NK cell antiviral responses can impede the spread and efficacy of OVs [115], which will be discussed in detail in the sections below.

### 5.3. NK Cell Memory Responses

NK cells were conventionally thought of as short lived and incapable of “memory” responses, which is typically associated with the adaptive immune system, but there is now considerable evidence supporting the existence of long-lived memory NK cells that develop in response to viral infection and cytokine stimulation [164,165,166,167], i.e., these NK cells exhibit quick and strong activation and cytotoxicity after a challenge from the same stimuli that they encountered before [164,167,168,169]. NK cell memory in response to viral infection is best understood using murine cytomegalovirus (MCMV) infection models. The long-lived NK cells, mainly a subset expressing the Ly49H receptor, exhibit rapid recall responses to an MCMV rechallenge. These NK cells undergo clonal expansion upon exposure to m157, a virally encoded protein, and are capable of controlling peripheral viremia [164]. Human NK cells can also develop memory. For example, the memory response against human cytomegalovirus (HCMV) is mediated by CD94/NKG2C^+^ NK cells and is dependent on CD2 co-stimulation. These NK cells are epigenetically distinct from naïve NK cells and quickly respond to an HCMV rechallenge, undergoing clonal expansion [170,171,172]. While the exact ligand promoting NK cell memory to HCMV infection remains unclear, various *UL40*-encoded peptides contribute to the recognition of HCMV-infected cells by NKG2C^+^ adaptive memory-like NK cells [173]. Antigen-specific NK cell responses to HIV and influenza have also been documented, with CD94/NKG2C^+^ NK cells recognizing peptides presented by HLA-E on infected cells [174].

The molecular mechanisms that regulate NK cell memory are not entirely unclear, and are, most likely, experimental-model dependent. However, recent studies have revealed some similarities between memory NK cells and memory T-cells. For instance, T-cell factor 1(Tcf-1) is a transcription factor which is associated with stemness and crucial for the development of T-cell memory, as well as progenitor exhausted T-cells [175,176,177,178,179,180,181]. Although its role in NK cells is known to limit granzyme B expression to prevent self-destruction [182], a recent study has shown that tissue-resident Tcf-1^+^ NK cells can promote enhanced recall responses against secondary infections [183]. Additionally, NK cells positive for Tcf-1 and Bach2, another transcription factor associated with stemness, exhibit memory-like responses in the context of zika virus and HIV infection [184,185]. These studies have demonstrated an expanded role of NK cells with stem-like features in response to infection, especially secondary infection.

In addition to memory response to infection, the simultaneous stimulation of NK cells with IL-12, IL-15, and IL-18 can lead to the generation of cytokine-induced memory-like (CIML) NK cells. CIML NK cells exhibit enhanced antitumor efficacy compared to naïve NK cells, and maintain improved functionality for up to four months post-stimulation [167,186]. CIML NK cells are highly effective as a cellular therapy for acute myeloid leukemia (AML), with promising results from multiple clinical trials. In a phase 1 clinical trial with the transfer of ex vivo IL-12/IL-15/IL-18-stimulated NK cells against pediatric AML patients, four out of eight patients were in complete remission within 28 days of treatment. Moreover, donor memory NK cells remained functional for 3 months post-implantation, contributing to durable remission in two patients [187]. Adoptive CIML NK cell-based therapies have been similarly effective in adult patients with relapsing/recurring AML, with four out of nine patients achieving complete remission [187,188,189,190]. Additionally, CIML NK cells have shown enhanced efficacy against solid tumors in both clinical and preclinical studies, indicating improved tumor control in patients with head and neck cancer, and ovarian cancer-bearing mice [191,192]. Intriguingly, tumor-induced memory-like NK cells (TIML) are formed during the tumor priming of NK cells. TIML NK cells display enhanced survival, more efficient immune synapse formation, and increased synthesis of perforin. TIML NK cells primed with leukemic cells of B cell precursor acute lymphoblastic leukemia and AML exhibited enhanced cytotoxicity against their respective cancers both in vitro and in vivo [193]. Collectively, NK cell memory holds the potential to enhance both antitumor and antiviral activity, while their role remains poorly elucidated in the context of cancer immunotherapy, particularly OVT.

## 6. NK Cell Biology in OVT

### 6.1. NK Cell Antitumor and Antiviral Responses in OVT

Several studies have demonstrated that OVs convert the immune-suppressive TME into an immune stimulatory condition, thereby improving the NK cell-mediated killing of tumor cells [116,117,118,194,195,196] (Table 1). Upon administration of OVs, NK cells effectively kill OV-infected cancer cells through their antiviral immune response. Additionally, OVT-mediated TME conversion reshapes the NK cell receptor profile, thereby increasing cytotoxicity against uninfected tumor cells [197] (Figure 2B). NK cells have also been shown to improve the efficacy of OVT through facilitating replication and localization of OVs. In the context of the oncolytic reovirus model, hyperactive NK cells in mice preconditioned with IL-2 and Treg-depleting antibody display increased extravasation, transmigration, and tumor infiltration, which led to increased reovirus localization [198]. Concordantly, oncolytic VSV replication and spreading in both in vitro and in vivo settings using B16 melanoma cells are facilitated by activated NK cells. Interestingly, NK cells are able to mediate the localization of systemically delivered oncolytic VSV to the sites of primary and metastasized tumors [199], as this is dampened by NK cell depletion [198,199]. The overall body of research on OVT suggests that NK cells have a substantial role in promoting OVT therapeutic efficacy.

Controversially, NK cells have also been reported to impede therapeutic outcomes of OVTs through their potent antiviral response [115,200] (Table 2). OVs upregulate the expression of ligands for NK cell activation receptors in infected cells, such as NKG2D ligands, MICA, and retinoic acid early transcript 1 alpha (RAE1α) [201,202], and many NCR ligands, including hemagglutinin and hemagglutinin-neuraminidase (HN) [203,204]. Additionally, OVs can lower MHCI expression in infected cells, increasing their vulnerability to NK cells [163]. NK cells have been reported to preferentially target virus-infected tumor cells over uninfected tumor cells through NKp30 and NKp46, causing early viral clearance, limiting the reservoirs of viral replication, and consequentially compromising therapeutic outcomes [115,205,206,207]. Notably, in experimental GBM mouse models, the antiviral responses of macrophage and microglia in the brain depend on NK cell activation, and NK cell depletion confers a significant survival advantage over a non-depleted group [115]. Similarly, NK cells dampen the efficacy of oncolytic VSV therapy against hepatocellular carcinoma [205,208]. The improved antitumoral efficacy of oncolytic VSV therapy is accomplished by equipping OVs with the equine herpes virus-1 glycoprotein G (gG_EHV-1_), which binds to a broad range of chemokines, thereby suppressing the chemotaxis of NK cells to the site of infection [205]. Furthermore, the beneficial effect of NK cell depletion on the antitumor efficacy of oHSV against GBM tumors has also been reported [200].

Although the premature clearance of OVs by NK cells possibly undermines the therapeutic outcomes of OVTs (Figure 1A), it is noteworthy that the antiviral responses of NK cells also serve a vital role in priming the adaptive immune system against tumors by promoting the recruitment and maturation of DCs [47,50,51,209,210,211,212]. The activated NK cells facilitate the recruitment of DCs to the TME through the release of chemokines, such as C-C motif chemokine ligand 5 (CCL5) and X-C motif chemokine ligand 1 (XCL1), which direct DC localization [212]. NK cells also release tumor necrosis factor alpha (TNF-α) and IFN-γ, promoting maturation of DCs and upregulating their TLR and MHCII expression [213,214,215,216], which consequently activate T-cells to initiate the adaptive antitumor response [217,218,219].

Based on the previous studies, mathematical models have been established to predict the therapeutic outcomes resulting from alterations to parameters, including initial OV infection, NK cell activation, and tumor burden [220,221]. These models extrapolate how NK cell activation affects the efficacy of OVT, either in a beneficial or detrimental way. High viral cytopathy and early antiviral NK cell responses are linked to the impediment of OVT, while the addition of combination therapies is linked to the enhancement of OVT efficacy. Although the role of NK cells in OVT is controversial and context-dependent, several studies have sought to enhance the potential of NK cells in OVT by either inhibiting antiviral NK cells responses or promoting antitumor responses.

### 6.2. Enhancing NK Cell Antitumor Responses

**Figure 3:**
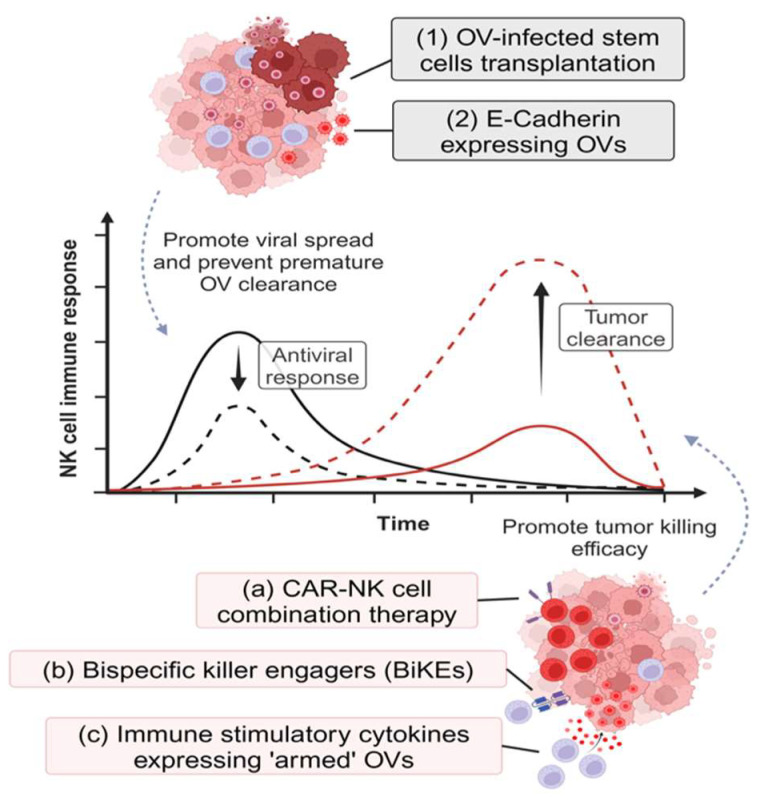
Strategies for improving OVT therapeutic efficacy. Improved therapeutic outcomes for OVT can be achieved by facilitating OV spread and/or promoting tumor-killing capacity of NK cells in different ways.

Although the role of NK cells in OVT has shown mixed outcomes, their potential to enhance the effectiveness of OVs has prompted the development of NK cell immunotherapies that can synergize with OVTs (Figure 3). The C-C chemokine receptor 5 (CCR5)–CCL5 chemotaxis pathway has been identified as a mechanism for NK cell infiltration into a tumor [222]. To capitalize on this interaction, Li et al. engineered NK cells to overexpress CCR5 and the oncolytic VV to express CCL5 [223]. These genetic modifications facilitate NK cell migration towards OV-infected tumor cells, but do not alter their tumor-killing efficacy their production of cytokines, such as IFN-γ and TNF-α. In in vivo settings, CCR5-engineered NK cells have reduced tumor size and prolonged mice survival in combination with CCL5-expressing OVs.

A combination of oHSV and epidermal growth factor receptor (EGFR)-specific CAR-NK92 cells has shown increased tumor-killing efficacy against breast cancer and brain metastatic cells, leading to better survival outcomes [224]. Similarly, an oHSV armed with the IL-15/IL-15Rα complex, which activates NK cells more efficiently than IL-15 alone [225,226,227,228], has been shown to increase the effectiveness of EGFR CAR-NK antitumor activity against GBM cell lines. The IL-15/IL-15Rα complex secreted from virus-infected tumor cells not only facilitates the recruitment of CAR-NK cells to the tumor site, but also promotes NK cell survival and priming [77,226,229,230]. In a study designed to test the effects of reovirus on DCs, NK cells co-cultured with DCs exposed to reovirus (reo-DCs) displayed enhanced tumor-killing efficacy compared to NK cells treated with IL-2 or NK cells co-cultured with untreated DCs. NK cells co-cultured with reo-DCs showed elevated IFN-γ secretion and, of note, reovirus exposure alone did not lead to the improved antitumor functionality of NK cells [66]. Treatment with bortezomib increased GBM sensitivity to adoptive NK cell therapy via upregulation of death receptor (DR)-5 [198]. This sensitivity was further enhanced by the addition of oHSV. The triple combination therapy of bortezomib, oHSV, and adoptive NK cell transfer has achieved superior responses against GBM in vitro and in vivo compared to bortezomib and NK cell adoptive transfer combination therapy [199]. Interestingly, the efficacy of oHSV and bortezomib treatment alone can be improved by endogenous NK depletion in GBM-bearing mice. This result indicates that the host NK cells may impede tumor clearance, and the adoptive transfer of NK cells is required for better therapeutic outcomes [200]. Therefore, a better understanding of the mechanisms of NK cell antitumor responses in the context of OVT has the potential to formulate combination therapies to improve the efficacy of OVT.

### 6.3. Inhibiting NK Cell Antiviral Responses

Several studies have aimed to prevent the premature clearance of viruses by NK cells while preserving their antitumor functionality (Figure 3). One such approach is to generate an oHSV expressing the cadherin 1 (*CDH1*) gene which encodes E-cadherin [231]. E-cadherin is a ligand of killer cell lectin-like receptor subfamily G, member 1 (KLRG1), expressed on terminally differentiated NK cells, and the binding of E-cadherin to KLRG1 impedes NK cell cytotoxicity [232,233]. In preclinical GBM models, CDH1-expressing OVs inhibit the cytotoxicity of KLRG1^+^ NK cells, which constitute more than 50% of the total NK cells within the TME. Compared to their parental oHSV, CDH1-expressing OVs have superior viral spread and are capable of improving overall survival in GBM-bearing mice [231]. Another approach has utilized “trojan horse” cell delivery strategies. These strategies utilize non-immunogenic cells as a reservoir of OV replication, functioning as a cellular therapy. For example, one study demonstrated that adipose-derived stem cells (ADSCs) inhibit NK cell-mediated antiviral responses, and yield superior viral spread when used as a delivery system for oncolytic VV [234]. However, for this strategy, MHC-matched donors are required to avoid graft rejection. A recent study demonstrated that it is possible to redirect the NK cell immune response towards the tumor. This is achieved by using an oncolytic measles virus that is genetically modified to express a bispecific killer engager (BiKE). BiKEs are capable of binding to two different targets. A BiKE targeting the carcinoembryonic antigen on the tumor cells and CD16 on the NK cells enables the formation of an immunological synapse between these cells, facilitating ADCC-mediated cancer cell clearance [235]. This approach increases NK cell reactivity against non-infected bystander tumor cells, preventing the early clearance of virus-infected cells.

## 7. Conclusions

In conclusion, the role of NK cells in OVT is pivotal. They have the potential to enhance OVT efficacy through their robust antitumor responses and have the capacity to prime the adaptive antitumor response. Conversely, they may impede OVT therapeutic outcomes by limiting viral replication and spread via their antiviral responses. The research addressing the optimal balance between these responses to maximize OVT efficacy is only just beginning, but the progress made thus far is promising. With ongoing research on new aspects of NK cell biology, such as NK cell memory, the synergy between OVT and NK cells will continue to improve.

## Figures and Tables

**Table 1:** NK cells contribute to therapeutic outcomes of OVTs in preclinical studies.

OV	Tumor Model	Combination Therapy	Study/References
(Measles virus) MV-GFP-HSNS-EGFRvIII	Glioma (GBM39)	Not applicable (N/A)	[116]
(Herpes simplex virus-1) gamma34.5 mutant	Melanoma lines (Syngeneic DBA/2)	N/A	[117]
(Vesicular stomatitis virus) VSV-ΔM-mp53	Melanoma lines (TS/A and B16)	N/A	[118]
(Reovirus) Reolysin	Leukemia cell line (K562)	N/A	[194]
Coxsackievirus B3	Non-small-cell lung carcinoma line (A549)	N/A	[195]
(Vaccinia virus) VVΔTKΔN1L-IL12	Lewis lung carcinoma	N/A	[196]
(Adenovirus) *dl*922-947, Ad3/Ad11p	Ovarian cancer (TOV21G and OVCAR4)	N/A	[197]
(Reovirus) WT type 3 Dearing strain	Melanoma line (B16)	Treg depletion and IL-2 treatment	[198]
(Vesicular stomatitis virus) VSV-GFP	Melanoma lines (B16 and YAC-1)	Treg depletion and IL-2 treatment	[199]

**Table 2:** NK cells impede therapeutic outcomes of OVTs in preclinical studies.

OV	Tumor Model	Combination Therapy	Study/References
(Herpes simplex virus-1) rQNestin34.5	Human glioma (U87dEGFR) Syngeneic mouse glioma (KR158dEGFR)	N/A	[115]
(Vesicular stomatitis virus) rVSV-gG and rVSV-f	Hepatocellular carcinoma lines (BHK-21, McA-RH7777)	N/A	[205]
(Herpes simplex virus-1) rQNestin34.5	Human GBM xenograft (GB30-FFL) Syngeneic mouse GBM (4C8)	TGF-β	[206]
(Herpes simplex virus-1) OV hrR3	Rat glioma line (D74/HveC)	cyclophosphamide (CPA)	[207]
(Vesicular stomatitis virus) rVSV-UL141	Hepatocellular carcinoma line (McA-RH7777)	N/A	[208]

## Abbreviations

ADAM	a disintegrin and metalloprotease
ADCC	antibody-dependent cellular cytotoxicity
ADSCs	adipose-derived stem cells
AML	acute myeloid leukemia
APCs	antigen-presenting cells
BAG6	BCL2-associated athanogene 6
BiKE	bispecific killer engager
CAR	chimeric antigen receptor
CCL5	C-C motif chemokine ligand 5
CCR5	C-C chemokine receptor 5
CDH1	cadherin 1
CI cycle	cancer-immunity cycle
CIML NK cells	cytokine-induced memory-like NK cells
CTLA-4	cytotoxic T lymphocyte-associated protein 4
DAMPs	damage-associated molecular pattern molecules
DCs	dendritic cells
DRR	durable response rate
EBV	Epstein Barr virus
ECM	extracellular matrix
EGFR	epidermal growth factor receptor
Erp5	endoplasmic reticulum protein 5
GBM	glioblastoma multiforme
gGEHV-1	equine herpes virus-1 glycoprotein G
GM-CSF	granulocyte–macrophage colony-stimulating factor
HCMV	human cytomegalovirus
HSP	heat shock protein
HN	hemagglutinin–neuraminidase
HPV	human papilloma virus
HSV	herpes simplex virus
ICB	immune checkpoint blockade
IDO	indoleamine 2:3-dioxygenase
IFN-γ	interferon-gamma
IL	interleukin
KIR	killer immunoglobulin-like receptors
KLRG1	killer cell lectin-like receptor subfamily G member 1
LILRB4	leukocyte immunoglobulin-like receptor subfamily B member 4
LCMV	lymphocytic choriomeningitis virus
MCMV	murine cytomegalovirus
MDSCs	myeloid-derived suppressor cells
MHC	major histocompatibility complex
MICA	MHC class I-related polypeptide sequence A
MMP14	matrix metallopeptidase 14
NCRs	natural cytotoxicity receptors
NCR3LG1	natural killer cell cytotoxicity receptor 3 ligand 1
NDV	Newcastle disease virus
NK cells	natural killer cells
oAds	oncolytic adenoviruses
oHSV	oncolytic herpes simplex virus
OVs	oncolytic viruses
OVT	oncolytic virotherapy
PAMPs	pathogen-associated molecular pattern molecules
PD-1	programmed cell death protein 1
PD-L1	programmed cell death ligand 1
PD-L2	programmed cell death ligand 2
PKR	protein kinase R
RAE1α	retinoic acid early transcript 1 alpha
scFv	single-chain variable fragment
TAAs	tumor-associated antigens
TAMs	tumor-associated macrophages
Tcf-1	T-cell factor 1
TGF	transforming growth factor
TIL	tumor-infiltrating lymphocytes
TLRs	toll-like receptors
TME	tumor microenvironment
Tregs	regulatory T-cells
TNF-α	tumor necrosis factor alpha
T-VEC	talimogene laherparepvec
VSV	vestibular stomatitis virus
VV	vaccinia virus
VZV	varicella zoster virus
XCL1	X-C motif chemokine ligand 1
